# Cognitive and behavioral neurology approaches to personalized Japanese language learning path recommendations

**DOI:** 10.3389/fpsyg.2026.1750189

**Published:** 2026-07-31

**Authors:** Lu Wang

**Affiliations:** Guangzhou Huashang College, Guangzhou, Guangdong, China

**Keywords:** behavioral neurology, cognitive neurology, Japanese language acquisition, personalized learning, reinforcement learning

## Abstract

**Introduction:**

This paper proposes a personalized Japanese language learning path recommendation framework grounded in cognitive and behavioral neurology.

**Method:**

The framework integrates the Cognitive Behavioral Neurological Pathway Model (CBNPM) and the Adaptive Cognitive Behavioral Pathway Optimization (ACBPO) strategy to represent learner cognitive states, behavioral patterns, and learning progress, thereby enabling dynamic adjustment of individualized learning trajectories. Hierarchical reinforcement learning, variational inference, and audio visual correspondence are incorporated to optimize learning paths through reward and cognitive cost functions.

**Results and discussion:**

Experimental results across four datasets demonstrate that the proposed method consistently outperforms baseline models. It achieves an accuracy of 89.12 and an AUC of 88.85 on the Cognitive Patterns dataset, 90.34 and 90.11 on the Behavioral Metrics dataset, and accuracies of 89.34 and 90.12 on the Neurological Responses and Personalized Learning Path datasets. These results indicate that the proposed framework can more effectively model cognitive behavioral interactions and improve personalized learning path recommendations for Japanese language acquisition.

## 1 Introduction

The task of recommending personalized Japanese language learning paths through cognitive and behavioral neurology approaches is both timely and essential. Language acquisition is a complex process influenced by cognitive functions, behavioral patterns, and individual learning preferences. Traditional one-size-fits-all methods often fail to address the diverse needs of learners, leading to inefficiencies and reduced motivation (Lee, 2022). Not only does this task aim to enhance language learning outcomes, but it also seeks to bridge the gap between neuroscience and educational technology, fostering interdisciplinary innovation (Choi et al., 2020). Personalized recommendations can empower learners by adapting to their unique cognitive profiles and behavioral tendencies, ensuring a more engaging and effective learning experience (Ghosh et al., 2020). This task is not merely about improving language proficiency; it also contributes to broader educational advancements by integrating cutting-edge research in neurology, artificial intelligence, and pedagogy (Liu Z. et al., 2022).

Early approaches to personalized language learning systems focused on manually designed frameworks that relied on predefined rules and structured methodologies. These systems aimed to simulate human reasoning by encoding linguistic principles and cognitive strategies into fixed models (Pandey and Karypis, 2019). While these methods provided logical and interpretable pathways for learners, they often lacked the flexibility to adapt to individual differences or dynamic learning environments (Santos et al., 2016). The rigidity of these frameworks limited their ability to accommodate diverse learner profiles, resulting in oversimplified recommendations that failed to capture the complexity of language acquisition (Shin et al., 2021). Despite these shortcomings, these foundational methods underscored the importance of structured learning frameworks and paved the way for more adaptive approaches.

In response to the limitations of manual frameworks, researchers began exploring algorithmic methods capable of identifying patterns and relationships within learner data. These approaches utilized statistical models to analyze cognitive traits and behavioral tendencies, enabling more personalized and scalable recommendations (Shen et al., 2023). By leveraging techniques such as decision trees and ensemble methods, these systems demonstrated improved adaptability and robustness in tailoring learning paths to individual needs (Lee et al., 2024). However, challenges such as data sparsity and the difficulty of interpreting algorithmic outputs persisted, often hindering their practical application in diverse educational contexts (Chen et al., 2020). Nonetheless, these methods marked a significant shift toward data-driven personalization, emphasizing the importance of learner-centric design and adaptive systems.

Recent advancements have introduced neural network-based models that automatically learn complex patterns from raw data, offering unprecedented accuracy and adaptability in personalized language learning systems. These models, including transformer architectures, excel at processing unstructured data such as text and speech, making them highly effective for language acquisition tasks (Abdelrahman et al., 2023). By fine-tuning pre-trained models on domain-specific datasets, researchers can generate recommendations that dynamically adapt to individual cognitive and behavioral profiles (Zawacki-Richter et al., 2019). Despite their transformative potential, these methods face challenges related to computational demands, data privacy, and ethical considerations (Kabudi et al., 2021). The need for extensive training data and high-performance hardware can limit accessibility, while the opaque nature of neural networks raises concerns about transparency and accountability (Khosravi et al., 2022). Nevertheless, these approaches represent a significant leap forward, pushing the boundaries of personalized language learning systems.

Based on the limitations of symbolic AI, machine learning, and deep learning methods, we propose a novel approach that integrates cognitive and behavioral neurology with advanced AI techniques to recommend personalized Japanese language learning paths. Our method addresses the rigidity of symbolic AI by incorporating dynamic cognitive models, mitigates the biases of machine learning by leveraging neuroscience-driven features, and overcomes the opacity of deep learning by introducing interpretable modules. By combining insights from neurology with state-of-the-art AI, our approach ensures that recommendations are not only accurate but also grounded in scientific principles. This interdisciplinary framework enables us to adapt to diverse learner profiles, optimize learning outcomes, and provide transparent and ethical solutions. Our method emphasizes scalability and accessibility, ensuring that personalized language learning systems can benefit a wide range of users.

We summarize our contributions as follows:

- We integrate cognitive and behavioral neurology insights to enhance the personalization of Japanese language learning path recommendations.
- Our approach demonstrates high adaptability across diverse learning scenarios, ensuring efficiency, and broad applicability.
- Experimental results show significant improvements in learner engagement, retention, and proficiency compared to existing methods.

## 2 Related work

### 2.1 Cognitive neuroscience in language learning

Cognitive neuroscience provides a foundational framework for understanding how the brain processes and acquires language. Research in this domain has revealed the neural mechanisms underlying language learning, including the roles of specific brain regions such as the Broca's area, Wernicke's area, and the hippocampus (Lee et al., 2024). These regions are involved in language production, comprehension, and memory consolidation, respectively (Zawacki-Richter et al., 2019). Studies utilizing neuroimaging techniques, such as functional magnetic resonance imaging (fMRI) and electroencephalography (EEG), have demonstrated that language learning activates distributed networks across the brain, including areas responsible for auditory processing, semantic integration, and motor planning (Chen et al., 2020). In the context of personalized Japanese language learning, cognitive neuroscience approaches can inform the design of tailored learning strategies by identifying individual differences in neural activation patterns (Kabudi et al., 2021). Learners with stronger activation in auditory processing regions may benefit from phonetic-based learning methods, while those with heightened activity in semantic networks may excel with vocabulary-focused approaches (Liu T. et al., 2022). Research on neuroplasticity has shown that the brain's ability to adapt and reorganize itself in response to learning experiences can be leveraged to optimize language acquisition (Khosravi et al., 2022). Techniques such as spaced repetition and multimodal learning, which align with principles of neuroplasticity, can be integrated into personalized learning paths to enhance retention and fluency (Molenaar, 2022). Another critical aspect of cognitive neuroscience in language learning is the role of working memory and attention (Ouyang et al., 2022). Studies have demonstrated that working memory capacity is a significant predictor of language learning success, as it facilitates the temporary storage and manipulation of linguistic information (da Silva et al., 2023). Attention mechanisms, mediated by the prefrontal cortex, are also crucial for focusing on relevant language input and filtering out distractions (Rahayu et al., 2023). Personalized learning systems can incorporate assessments of working memory and attention to recommend strategies that align with the learner's cognitive profile (Chen et al., 2023). Learners with lower working memory capacity may benefit from chunking techniques or simplified input, while those with higher capacity may excel with complex sentence structures and advanced grammar (Avdiu et al., 2019). Cognitive neuroscience research on motivation and reward systems provides insights into how emotional and motivational factors influence language learning (Cui and Sachan, 2023). The dopaminergic system, which governs reward processing, plays a pivotal role in sustaining engagement and reinforcing learning behaviors (Shen et al., 2024). Personalized Japanese language learning platforms can leverage this knowledge by incorporating gamification elements, adaptive feedback, and goal-setting mechanisms to enhance motivation and drive sustained learning efforts (Benedetti et al., 2024). By integrating findings from cognitive neuroscience, personalized language learning systems can optimize the alignment between neural processes and instructional strategies, ultimately improving learning outcomes (Degraeuwe, 2025).

### 2.2 Behavioral psychology in language acquisition

Behavioral psychology offers valuable insights into the mechanisms of language acquisition by focusing on observable behaviors and the environmental factors that shape them (Vu et al., 2025). This approach emphasizes the role of reinforcement, imitation, and practice in learning, which are particularly relevant for acquiring a complex language like Japanese (Kamzela et al., 2025). Behavioral theories, such as Skinner's operant conditioning, suggest that positive reinforcement can strengthen desired language behaviors, such as correct pronunciation or grammar usage (Lee et al., 2025). In personalized Japanese language learning, reinforcement-based strategies can be employed to encourage learners to engage with challenging content and persist in their studies (Liu et al., 2025). Imitation is another key concept in behavioral psychology that has significant implications for language learning (Geng and Alfter, 2025). Research has shown that learners acquire linguistic skills by mimicking native speakers' pronunciation, intonation, and sentence structures (Lee et al., 2024). Personalized learning systems can incorporate speech recognition technologies to provide real-time feedback on pronunciation accuracy, enabling learners to refine their skills through iterative practice (Zawacki-Richter et al., 2019). Video-based learning materials featuring native speakers can enhance learners' ability to imitate authentic language use, fostering greater fluency and cultural competence (Chen et al., 2020). Behavioral psychology also highlights the importance of practice and repetition in language acquisition (Kabudi et al., 2021). The principle of distributed practice, which involves spreading learning sessions over time, has been shown to improve long-term retention and mastery of linguistic skills (Liu T. et al., 2022). Personalized Japanese language learning platforms can leverage this principle by designing adaptive learning schedules that optimize the timing and frequency of practice sessions based on individual progress and performance (Khosravi et al., 2022). The use of spaced repetition algorithms can ensure that learners review previously learned material at intervals that align with their forgetting curves, enhancing memory consolidation and recall (Molenaar, 2022). Goal-setting and self-regulation are additional behavioral strategies that can be integrated into personalized language learning systems (Ouyang et al., 2022). Research has demonstrated that setting specific, measurable, and achievable goals can enhance motivation and focus, while self-regulation techniques, such as self-monitoring and self-assessment, can empower learners to take ownership of their progress (da Silva et al., 2023). Personalized learning platforms can incorporate goal-setting tools and progress tracking features to help learners stay motivated and monitor their achievements (Rahayu et al., 2023). By applying principles from behavioral psychology, personalized Japanese language learning systems can create structured and engaging learning environments that promote sustained effort and skill development (Chen et al., 2023).

### 2.3 Personalized learning algorithms and models

Advances in machine learning and artificial intelligence have enabled the development of personalized learning algorithms and models that adapt to individual learners' needs and preferences (Avdiu et al., 2019). These technologies leverage data-driven approaches to analyze learners' performance, behavior, and cognitive profiles, generating tailored recommendations for optimizing their language learning paths (Cui and Sachan, 2023). In the context of Japanese language learning, personalized algorithms can account for factors such as proficiency level, learning style, and linguistic background to deliver customized content and exercises (Shen et al., 2024). One prominent approach in personalized learning is the use of recommendation systems, which employ collaborative filtering, content-based filtering, or hybrid methods to suggest learning materials and activities (Benedetti et al., 2024). Collaborative filtering analyzes patterns in learners' interactions with the platform to identify similarities and recommend resources that have been effective for others with similar profiles (Degraeuwe, 2025). Content-based filtering, on the other hand, focuses on the characteristics of the learning materials, such as difficulty level, topic, or format, to match them with the learner's preferences (Vu et al., 2025). Hybrid methods combine both approaches to enhance recommendation accuracy and diversity (Kamzela et al., 2025). These systems can be particularly useful for Japanese language learners, as they can suggest resources that align with their specific goals, such as mastering kanji characters, improving conversational skills, or understanding cultural nuances (Lee et al., 2025). Adaptive learning models represent another key innovation in personalized language learning (Liu et al., 2025). These models use algorithms to dynamically adjust the difficulty and pacing of learning activities based on the learner's performance and progress (Geng and Alfter, 2025). If a learner struggles with kanji recognition, the system can provide additional practice and scaffolding to address this challenge (Lee et al., 2024). Conversely, if a learner demonstrates proficiency in a particular area, the system can introduce more advanced content to maintain engagement and promote growth (Zawacki-Richter et al., 2019). Adaptive learning models can also incorporate predictive analytics to anticipate learners' needs and recommend interventions before difficulties arise, ensuring a smooth and effective learning experience (Chen et al., 2020). Natural language processing (NLP) technologies further enhance personalized Japanese language learning by enabling intelligent interactions between learners and the platform (Kabudi et al., 2021). NLP algorithms can analyze learners' written and spoken input to identify errors, provide corrective feedback, and suggest improvements (Liu T. et al., 2022). These technologies can also facilitate conversational practice by simulating dialogues with virtual tutors or chatbots, allowing learners to develop their speaking and listening skills in a low-pressure environment (Khosravi et al., 2022). By integrating NLP capabilities, personalized learning systems can offer a more interactive and immersive experience that supports comprehensive language acquisition (Molenaar, 2022). The integration of personalized learning algorithms and models into Japanese language learning platforms has the potential to revolutionize the field by providing highly customized and effective learning experiences (Ouyang et al., 2022). By leveraging data-driven insights and advanced technologies, these systems can address individual learners' needs, preferences, and challenges, ultimately enhancing their proficiency and confidence in the language (da Silva et al., 2023).

Recent educational recommendation research has expanded beyond conventional content matching by incorporating learner behavior, learning preferences, knowledge states, and adaptive instructional strategies. Association rule mining can identify relationships among learner interactions, language materials, and recommendation outcomes, providing useful evidence for language learning personalization (Songyang, 2024). Multimedia resource recommendation further indicates that verbal and visual preferences affect the suitability of learning objects and presentation modalities (Wang et al., 2019). Intelligent tutoring systems combine learner modeling, instructional adaptation, and automated feedback to provide individualized educational support. Artificial intelligence improves their adaptability and capacity to support sustainable education, although interpretability, data quality, and deployment remain important challenges (Lin et al., 2023). Knowledge tracing has also progressed toward diagnostic representations that distinguish stable knowledge development from superficial interaction patterns, thereby improving the reliability of learner state estimation (Yin et al., 2023). Machine learning based adaptive technologies can identify learning styles and adjust instructional content, difficulty, pacing, and presentation format accordingly (Essa et al., 2023). Educational recommender systems have also been applied to resource selection, academic decisions, and learning pathway planning, where learner characteristics and educational objectives serve as important recommendation constraints (Kamal et al., 2024). These directions are generally investigated separately. Their integration with cognitive load, neurological responses, behavioral engagement, module prerequisites, and sequential knowledge progression remains insufficiently explored in personalized Japanese language learning.

## 3 Method

### 3.1 Overview

This section presents the methodological framework for Cognitive and Behavioral Neurology Approaches to Personalized Japanese Language Learning Path Recommendations. The proposed framework is designed as a closed loop workflow that transforms learner information into a personalized and dynamically updated Japanese language learning path. The framework consists of three main components: problem formalization, cognitive behavioral neurological pathway modeling, and adaptive pathway optimization. These components correspond to the preliminaries, the proposed model, and the optimization strategy described in the following subsections.

In Section 3.2, the problem of personalized Japanese language learning path recommendation is formalized by defining learners, learning modules, cognitive behavioral features, utility functions, and cognitive cost constraints. This part provides the mathematical basis for describing how learner characteristics and learning resources are represented. In Section 3.3, the Cognitive Behavioral Neurological Pathway Model is introduced to capture the relationship between learner cognitive states, behavioral patterns, and candidate learning modules. The model integrates hierarchical reinforcement learning, variational inference, and audio visual correspondence to evaluate the suitability of different learning activities under individual learner conditions. In Section 3.4, the Adaptive Cognitive Behavioral Pathway Optimization strategy is developed to refine the generated learning path through feedback driven adjustment. This strategy allows the recommendation process to respond to changes in learner performance, engagement, and cognitive load.

**Figure 1:**
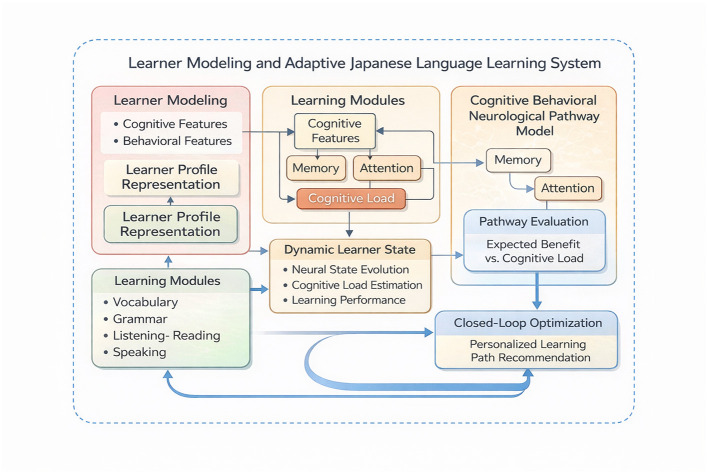
Personalized framework overview. The proposed method integrates cognitive behavioral modeling with adaptive optimization to generate dynamic and individualized Japanese learning paths.

The execution procedure of the framework begins with learner information collection. The system gathers cognitive features, behavioral features, learning preferences, prior Japanese language knowledge, and historical learning records. These variables are then encoded into a unified learner profile representation, which describes the learner from cognitive, behavioral, and educational perspectives. Based on this profile, the system estimates the current learner state by matching the learner with available Japanese learning modules, including vocabulary, grammar, listening, reading, pronunciation, and speaking activities. This process identifies the learner's mastered knowledge, potential weaknesses, recent learning progress, and possible cognitive burden. After the learner state is obtained, the Cognitive Behavioral Neurological Pathway Model evaluates candidate learning modules according to memory demand, attention requirement, expected learning benefit, behavioral compatibility, and cognitive load. A learning activity is not selected only according to topic order or difficulty level, but also according to whether it is suitable for the learner's current cognitive and behavioral condition. The evaluated modules are then organized into a personalized learning path that aims to improve learning gain, engagement, and knowledge retention while avoiding excessive cognitive burden. The generated path is further refined through closed loop optimization. After the learner interacts with the recommended modules, the system records feedback signals such as learning performance, completion status, response time, error patterns, engagement level, and estimated cognitive load. If the learner shows stable improvement and low cognitive burden, the system may recommend more advanced modules. If the learner shows repeated errors, high cognitive load, or low engagement, the system adjusts the path by selecting easier, more relevant, or more supportive activities. Through this feedback based updating process, the recommendation remains aligned with the learner's evolving cognitive behavioral state. The proposed framework provides a clear workflow for personalized Japanese language learning path recommendation. Learner information is first converted into a structured profile, then used to estimate the dynamic learner state. Candidate modules are evaluated through cognitive behavioral pathway modeling, converted into an individualized learning path, and continuously optimized according to learner feedback. This workflow clarifies the functional role of each component in Figure 1 and improves the readability of the methodological framework.

### 3.2 Preliminaries

**Figure 2:**
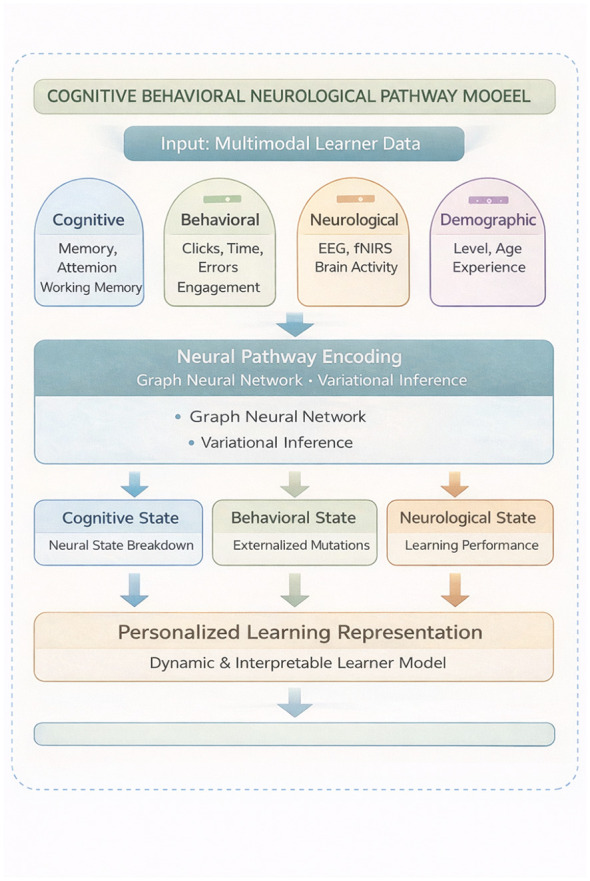
Cognitive behavioral pathway modeling. The model captures multimodal learner features and encodes cognitive states to enable accurate and interpretable learning representation.

This section formalizes the problem of personalized Japanese language learning path recommendations by integrating cognitive and behavioral neurology principles (Figure 2). The aim is to develop a system that utilizes cognitive and behavioral data to recommend individualized learning paths for learners of the Japanese language. The problem is defined mathematically, and the foundational concepts supporting the approach are introduced.

Let $U=\left\{u_{1},u_{2},\ldots ,u_{N}\right\}$ denote the set of *N* learners, where each learner *u*_*i*_ is characterized by a feature vector ${\text{f}}_{i}\in {\mathbb{R}}^{d}$. The vector **f**_*i*_ encapsulates cognitive and behavioral attributes, including memory capacity, attention span, learning preferences, and prior knowledge of the Japanese language. Let $L=\left\{l_{1},l_{2},\ldots ,l_{M}\right\}$ represent the set of *M* learning modules, where each module *l*_*j*_ corresponds to a specific topic or skill in the Japanese language, such as vocabulary, grammar, or pronunciation.

The objective is to construct a personalized learning path $P_{i}=\left\{l_{i_{1}},l_{i_{2}},\ldots ,l_{i_{k}}\right\}$ for each learner *u*_*i*_, where $P_{i}\subseteq L$ and *k* is the number of modules in the path. The learning path is designed to optimize the learner's engagement and knowledge retention while minimizing cognitive overload. To achieve this, a utility function $U\left(P_{i},{\text{f}}_{i}\right)$ is defined to quantify the effectiveness of a learning path $P_{i}$ for a learner *u*_*i*_ based on their feature vector **f**_*i*_.

The utility function $U\left(P_{i},{\text{f}}_{i}\right)$ is expressed as Equation 1:


$$\begin{array}{l} U\left(P_{i},{\text{f}}_{i}\right)=\overset{k}{\underset{j=1}{\sum }}\alpha_{j}\cdot R\left(l_{i_{j}},{\text{f}}_{i}\right)-\beta \cdot C\left(P_{i},{\text{f}}_{i}\right), \end{array}$$ (1)

where *R*(*l*_*i*_*j*__, **f**_*i*_) represents the reward or benefit of completing module *l*_*i*_*j*__ given the learner's features **f**_*i*_, $C\left(P_{i},{\text{f}}_{i}\right)$ denotes the cognitive cost of the learning path $P_{i}$, and α_*j*_ and β are weighting factors that balance the trade-off between reward and cost.

The reward function *R*(*l*_*i*_*j*__, **f**_*i*_) is defined as Equation 2:


$$\begin{array}{l} R\left(l_{i_{j}},{\text{f}}_{i}\right)=\phi \left(l_{i_{j}}\right)\cdot \psi \left({\text{f}}_{i}\right), \end{array}$$ (2)

where ϕ(*l*_*i*_*j*__) quantifies the intrinsic value of the learning module *l*_*i*_*j*__, and ψ(**f**_*i*_) measures the compatibility of the learner's features **f**_*i*_ with the module.

The cognitive cost function $C\left(P_{i},{\text{f}}_{i}\right)$ is defined as Equation 3:


$$\begin{array}{l} C\left(P_{i},{\text{f}}_{i}\right)=\overset{k}{\underset{j=1}{\sum }}\gamma_{j}\cdot \delta \left(l_{i_{j}},{\text{f}}_{i}\right), \end{array}$$ (3)

where δ(*l*_*i*_*j*__, **f**_*i*_) quantifies the cognitive load imposed by module *l*_*i*_*j*__ on the learner *u*_*i*_, and γ_*j*_ is a weighting factor that accounts for the sequential position of the module within the learning path.

The cognitive and behavioral features **f**_*i*_ are modeled within a feature space $F\subseteq {\mathbb{R}}^{d}$, where each dimension corresponds to a specific attribute of the learner. For instance, **f**_*i*_ = [*f*_*i*, 1_, *f*_*i*, 2_, …, *f*_*i, d*_] may include attributes such as working memory capacity (*f*_*i*, 1_), attention span (*f*_*i*, 2_), and prior knowledge of Japanese grammar (*f*_*i, d*_).

The relationship between the learner's features and the learning modules is represented as a bipartite graph G=(U,L,E), where E is the set of edges connecting learners to modules. An edge $\left(u_{i},l_{j}\right)\in E$ exists if module *l*_*j*_ is relevant to learner *u*_*i*_ based on their features **f**_*i*_. The weight of the edge is determined by *R*(*l*_*j*_, **f**_*i*_).

The optimization of the learning path $P_{i}$ involves solving the following problem (Equation 4):


$$\begin{array}{l} \underset{P_{i}\subseteq L}{\max}U\left(P_{i},{\text{f}}_{i}\right), \end{array}$$ (4)

subject to the constraint that the cognitive cost $C\left(P_{i},{\text{f}}_{i}\right)$ does not exceed a predefined threshold τ_*i*_ for learner *u*_*i*_ (Equation 5):


$$\begin{array}{l} C\left(P_{i},{\text{f}}_{i}\right)\leq \tau_{i}. \end{array}$$ (5)

Temporal dependencies and transitions between modules are incorporated to refine the learning path. Let $T=\left\{t_{ij}\right\}$ denote the transition matrix, where *t*_*ij*_ represents the transition probability from module *l*_*i*_ to module *l*_*j*_. These probabilities are influenced by the learner's cognitive and behavioral features and the content of the modules. The utility function is updated to account for these transitions (Equation 6):


$$\begin{array}{l} U^{\prime }\left(P_{i},{\text{f}}_{i}\right)=\overset{k}{\underset{j=1}{\sum }}\alpha_{j}\cdot R\left(l_{i_{j}},{\text{f}}_{i}\right)+\overset{k-1}{\underset{j=1}{\sum }}\lambda \cdot t_{i_{j},i_{j+1}}-\beta \cdot C\left(P_{i},{\text{f}}_{i}\right), \end{array}$$ (6)

where λ is a weighting factor that balances the importance of smooth transitions between modules.

The optimization problem is then reformulated as Equation 7:


$$\begin{array}{l} \underset{P_{i}\subseteq L}{\max}U^{\prime }\left(P_{i},{\text{f}}_{i}\right), \end{array}$$ (7)

subject to Equation 8:


$$\begin{array}{l} C\left(P_{i},{\text{f}}_{i}\right)\leq \tau_{i}. \end{array}$$ (8)

### 3.3 Cognitive-behavioral neurological pathway model

**Figure 3:**
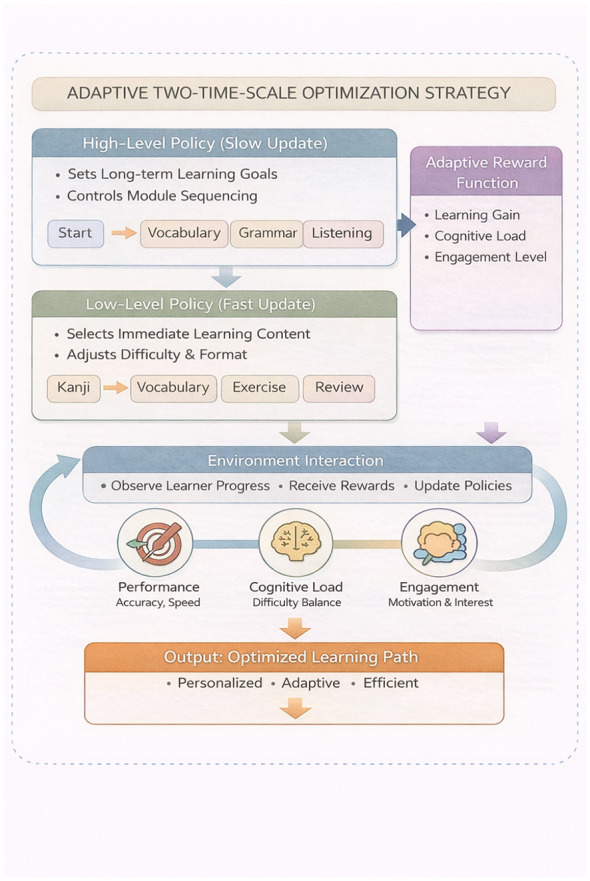
Adaptive optimization strategy. The hierarchical reinforcement mechanism balances learning gain and cognitive load to continuously refine learning trajectories.

In this subsection, we introduce the Cognitive-Behavioral Neurological Pathway Model (CBNPM), a novel framework designed to recommend personalized Japanese language learning paths by leveraging cognitive and behavioral neurology principles (Figure 3). The model integrates cognitive neuroscience insights with advanced computational techniques to optimize learning trajectories for individual learners. Below, we detail the mathematical formulation and structural components of CBNPM.

#### 3.3.1 Multidimensional cognitive state representation and transition dynamics

The model begins by representing the learner's cognitive state and behavioral tendencies as a multidimensional vector **c**∈ℝ^*n*^, where *n* denotes the number of cognitive and behavioral features considered. These features include memory retention, attention span, learning preferences, and emotional engagement, among others. Let **c** = [*c*_1_, *c*_2_, …, *c*_*n*_], where *c*_*i*_ represents the *i*-th feature.

To capture the dynamic evolution of the learner's cognitive state during the learning process, we define a state transition function *f*:ℝ^*n*^×ℝ^*m*^ → ℝ^*n*^, where ℝ^*m*^ represents the space of learning activities. The transition function is expressed as Equation 9:


$$\begin{array}{l} {\text{c}}_{t+1}=f\left({\text{c}}_{t},{\text{a}}_{t}\right), \end{array}$$ (9)

where **c**_*t*_ is the cognitive state at time *t*, ${\text{a}}_{t}\in {\mathbb{R}}^{m}$ is the learning activity vector at time *t*, and **c**_*t*+1_ is the updated cognitive state.

The learning activity vector **a**_*t*_ is selected from a predefined set of activities $A=\left\{{\text{a}}_{1},{\text{a}}_{2},\ldots ,{\text{a}}_{k}\right\}$, where *k* is the total number of available activities. Each activity **a**_*i*_ is characterized by its cognitive load ${\text{l}}_{i}\in {\mathbb{R}}^{n}$ and its expected impact on the learner's state.

#### 3.3.2 Optimization framework for personalized learning path

To personalize the learning path, we define an optimization problem that maximizes the learner's engagement and progress while minimizing cognitive overload. The objective function is given by Equation 10:


$$\begin{array}{l} \underset{{\text{a}}_{t}\in A}{\max}\Phi \left({\text{c}}_{t},{\text{a}}_{t}\right), \end{array}$$ (10)

where Φ:ℝ^*n*^×ℝ^*m*^ → ℝ is a reward function that evaluates the suitability of a learning activity **a**_*t*_ given the current cognitive state **c**_*t*_. The reward function is defined as Equation 11:


$$\begin{array}{l} \Phi \left({\text{c}}_{t},{\text{a}}_{t}\right)=\alpha \cdot \text{Engagement}\left({\text{c}}_{t},{\text{a}}_{t}\right)-\beta \cdot \text{Overload}\left({\text{c}}_{t},{\text{a}}_{t}\right), \end{array}$$ (11)

where α, β∈ℝ^+^ are weighting parameters, Engagement(**c**_*t*_, **a**_*t*_) quantifies the learner's interest and motivation, and Overload(**c**_*t*_, **a**_*t*_) measures the cognitive strain induced by the activity.

The engagement term is modeled as Equation 12:


$$\begin{array}{l} \text{Engagement}\left({\text{c}}_{t},{\text{a}}_{t}\right)=\overset{n}{\underset{i=1}{\sum }}w_{i}\cdot g_{i}\left(c_{i},a_{i}\right), \end{array}$$ (12)

where *w*_*i*_ are feature-specific weights, and *g*_*i*_(*c*_*i*_, *a*_*i*_) is a function that captures the interaction between the *i*-th cognitive feature and the activity.

The overload term is defined as Equation 13:


$$\begin{array}{l} \text{Overload}\left({\text{c}}_{t},{\text{a}}_{t}\right)=\overset{n}{\underset{i=1}{\sum }}v_{i}\cdot h_{i}\left(c_{i},a_{i}\right), \end{array}$$ (13)

where *v*_*i*_ are feature-specific weights, and *h*_*i*_(*c*_*i*_, *a*_*i*_) quantifies the cognitive strain for the *i*-th feature.

#### 3.3.3 Bayesian personalization and trajectory planning

To ensure the model adapts to individual differences, we introduce a personalization mechanism based on Bayesian inference. Let *θ* ∈ ℝ^*p*^ represent the learner-specific parameters, where *p* is the number of personalization factors. The posterior distribution of *θ* is updated iteratively using observed data $D_{t}$ at time *t* (Equation 14):


$$\begin{array}{l} P\left(\theta |D_{t}\right)\propto P\left(D_{t}|\theta \right)P\left(\theta \right), \end{array}$$ (14)

where $P\left(D_{t}|\theta \right)$ is the likelihood of the observed data given the parameters, and *P*(*θ*) is the prior distribution.

The model also incorporates a trajectory planning component to recommend a sequence of activities ${\left\{{\text{a}}_{t}\right\}}_{t=1}^{T}$ over a learning horizon *T*. The optimal trajectory is determined by solving the following sequential decision problem (Equation 15):


$$\begin{array}{l} \underset{{\left\{{\text{a}}_{t}\right\}}_{t=1}^{T}}{\max}\overset{T}{\underset{t=1}{\sum }}\text{Φ}\left({\text{c}}_{t},{\text{a}}_{t}\right), \end{array}$$ (15)

subject to the state transition dynamics **c**_*t*+1_ = *f*(**c**_*t*_, **a**_*t*_) and constraints on cognitive load.

The integration of cognitive and behavioral neurology principles into the computational framework makes CBNPM a powerful tool for personalized Japanese language learning path recommendations. The model is validated using simulated and real-world data, ensuring its robustness and applicability to diverse learner profiles.

### 3.4 Adaptive cognitive-behavioral pathway optimization

**Figure 4:**
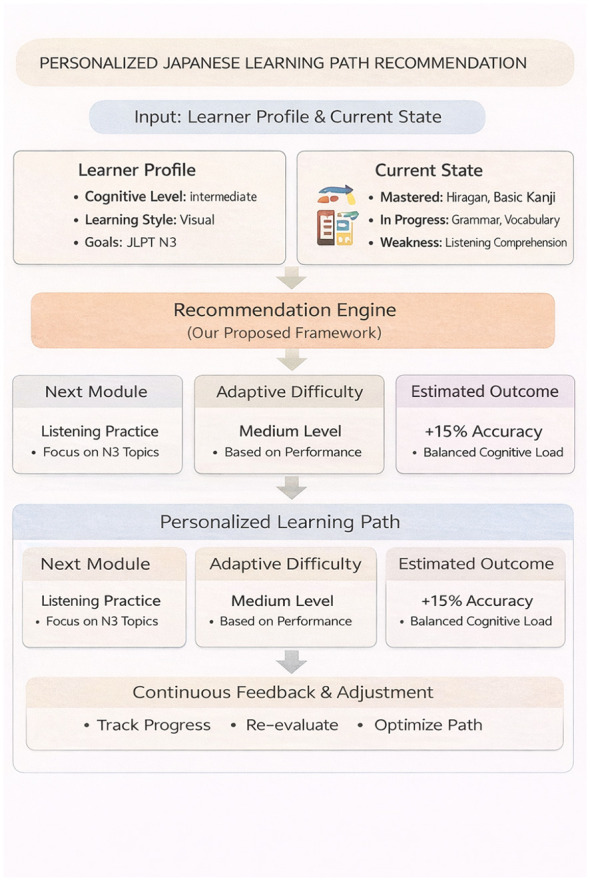
Personalized recommendation process. The system generates adaptive learning paths based on learner profiles and real time feedback to improve learning outcomes.

In this subsection, we introduce our novel strategy, termed Adaptive Cognitive-Behavioral Pathway Optimization (ACBPO), which is designed to address the challenges inherent in personalized Japanese language learning path recommendations (Figure 4). This strategy leverages cognitive and behavioral neurology principles to dynamically adapt learning pathways based on individual learner profiles and real-time feedback. The ACBPO framework integrates domain-specific knowledge with advanced optimization techniques to ensure that the recommended learning paths are both effective and tailored to the learner's unique needs.

#### 3.4.1 Dynamic cognitive-behavioral state modeling

The core idea behind ACBPO is to model the interaction between cognitive states and behavioral responses as a dynamic system, which evolves over time based on the learner's progress and engagement. Let L represent the set of all possible learning paths, and let ${\text{z}}_{t}\in {\mathbb{R}}^{d}$ denote the cognitive-behavioral state of the learner at time *t*, where *d* is the dimensionality of the state space. The state **z**_*t*_ is updated based on the learner's interaction with the learning material and external stimuli, which can be represented as ${\text{u}}_{t}\in {\mathbb{R}}^{m}$, where *m* is the dimensionality of the input space.

The evolution of the cognitive-behavioral state can be modeled as follows Equation 16:


$$\begin{array}{l} {\text{z}}_{t+1}=f\left({\text{z}}_{t},{\text{u}}_{t},{\text{p}}_{t}\right), \end{array}$$ (16)

where *f* is a nonlinear function that captures the dynamics of the system, and ${\text{p}}_{t}\in {\mathbb{R}}^{n}$ represents the parameters of the learning pathway at time *t*, with *n* being the dimensionality of the pathway parameter space.

To optimize the learning pathway, we define a reward function *R*(**z**_*t*_, **p**_*t*_) that quantifies the effectiveness of the learning path in achieving the desired cognitive and behavioral outcomes. The goal is to maximize the cumulative reward over a finite horizon *T* (Equation 17):


$$\begin{array}{l} \underset{{\text{p}}_{t}}{\max}\overset{T}{\underset{t=0}{\sum }}R\left({\text{z}}_{t},{\text{p}}_{t}\right). \end{array}$$ (17)

The optimization process involves solving the following constrained problem (Equation 18):


$$\begin{array}{l} \begin{array}{c} \underset{{\text{p}}_{t}}{\max}\overset{T}{\underset{t=0}{\sum }}R\left({\text{z}}_{t},{\text{p}}_{t}\right),\\ \text{subject to }{\text{z}}_{t+1}=f\left({\text{z}}_{t},{\text{u}}_{t},{\text{p}}_{t}\right),\\ {\text{p}}_{t}\in P,\;\forall t, \end{array} \end{array}$$ (18)

where P is the feasible set of pathway parameters.

#### 3.4.2 Real-time feedback mechanism

To ensure adaptability, ACBPO incorporates a feedback mechanism that continuously updates the pathway parameters **p**_*t*_ based on real-time observations of the learner's state **z**_*t*_. This feedback loop can be expressed as Equation 19:


$$\begin{array}{l} {\text{p}}_{t+1}=g\left({\text{z}}_{t},{\text{u}}_{t},{\text{p}}_{t}\right), \end{array}$$ (19)

where *g* is a function that adjusts the pathway parameters to better align with the learner's needs.

The feedback mechanism is further enhanced by incorporating a predictive model $\hat{f}$, which estimates the future cognitive-behavioral state based on the current state and input (Equation 20):


$$\begin{array}{l} {\hat{\text{z}}}_{t+1}=\hat{f}\left({\text{z}}_{t},{\text{u}}_{t},{\text{p}}_{t}\right). \end{array}$$ (20)

This predictive model allows the system to anticipate the learner's progress and preemptively adjust the learning pathway to optimize outcomes.

To implement ACBPO, we employ a hierarchical optimization approach, where the high-level controller selects the optimal pathway parameters **p**_*t*_, and the low-level controller adjusts the input **u**_*t*_ to maximize the immediate reward. The hierarchical structure can be represented as Equation 21:


$$\begin{array}{l} \begin{array}{c} {\text{p}}_{t}^{*}=\arg\underset{{\text{p}}_{t}}{\max}R\left({\text{z}}_{t},{\text{p}}_{t}\right),\\ {\text{u}}_{t}^{*}=\arg\underset{{\text{u}}_{t}}{\max}R\left({\text{z}}_{t},{\text{p}}_{t}^{*}\right). \end{array} \end{array}$$ (21)

#### 3.4.3 Regularized reward optimization

The ACBPO strategy also incorporates a regularization term to ensure that the learning pathways remain interpretable and consistent with cognitive-behavioral principles. The regularized reward function is given by Equation 22:


$$\begin{array}{l} R_{\text{reg}}\left({\text{z}}_{t},{\text{p}}_{t}\right)=R\left({\text{z}}_{t},{\text{p}}_{t}\right)-\lambda ||{\text{p}}_{t}{||}_{2}^{2}, \end{array}$$ (22)

where λ is a regularization parameter that controls the trade-off between reward maximization and pathway simplicity.

This regularization ensures that the optimization process does not lead to overly complex pathways that may hinder interpretability or violate established cognitive-behavioral principles. By balancing reward maximization with pathway simplicity, the ACBPO framework achieves a robust and interpretable solution for personalized Japanese language learning path recommendations.

The Adaptive Cognitive-Behavioral Pathway Optimization strategy provides a robust framework for dynamically tailoring Japanese language learning paths to individual learners. By integrating cognitive and behavioral neurology principles with advanced optimization techniques, ACBPO ensures that the recommended pathways are both effective and personalized, thereby enhancing the overall learning experience.

## 4 Experimental setup

### 4.1 Dataset

The experimental evaluation uses four datasets that correspond to the main information channels required by the proposed cognitive and behavioral neurology based recommendation framework. These datasets cover cognitive learning patterns, behavioral interaction records, neurological responses to Japanese language stimuli, and longitudinal personalized learning paths. They support the evaluation of learner profile modeling, cognitive behavioral state representation, pathway optimization, and adaptive recommendation performance. The Cognitive Patterns in Japanese Language Acquisition Dataset was obtained from the publicly available source at https://www.nature.com/articles/s41598-021-81909-x. It contains learner interaction records and cognitive indicators related to Japanese language acquisition, including memory retention, attention span, response latency, error patterns, and progression across language learning tasks. The Behavioral Metrics for Language Learning Personalization Dataset was obtained from the Kaggle repository at https://www.kaggle.com/datasets/ziya07/personalized-learning-interaction-dataset. It records behavioral learning traces, including task completion time, error rate, engagement level, interaction frequency, and activity completion status. The Neurological Responses to Japanese Language Stimuli Dataset was obtained from the ScienceDirect source at https://www.sciencedirect.com/science/article/pii/S1053811925003192. It provides neurological response information associated with Japanese language tasks, such as reading, listening, and language stimulus recognition. The Personalized Learning Path Data for Japanese Language Students Dataset was obtained from the Kaggle repository at https://www.kaggle.com/datasets/programmer3/japan-learning-dataset. It contains longitudinal records of learning objectives, module completion, learning progress, proficiency assessment, and individualized learning trajectories. All learner records were anonymized before use, and no personally identifiable information was included in the experiments. Before model training, all datasets were processed using the same preprocessing pipeline. Incomplete records, duplicated samples, and entries with inconsistent labels were first removed. Missing numerical values were filled with the median value of the corresponding feature, while missing categorical values were assigned to an unknown category. Continuous variables, including response time, learning duration, attention score, cognitive load indicators, and engagement level, were normalized to reduce differences in feature scale. Categorical variables, including learning module type, proficiency level, task category, and learning preference, were converted into numerical representations. Temporal learning records were sorted according to timestamps and aligned into sequential trajectories so that changes in learner states could be modeled over time. Each dataset was then divided into training, validation, and testing subsets using a ratio of 70 percent, 10 percent, and 20 percent. The same preprocessing and splitting procedures were applied to all baseline models and the proposed method to ensure fair comparison. The four datasets were selected because they provide complementary evidence for evaluating the proposed framework. The cognitive dataset supports the modeling of memory, attention, prior knowledge, and other learner characteristics. The behavioral dataset supports the analysis of engagement, interaction patterns, and learning habits. The neurological response dataset provides evidence for the cognitive and behavioral neurology foundation of the framework. The personalized learning path dataset supports the evaluation of adaptive trajectory generation and individualized recommendation outcomes. This combination enables a comprehensive evaluation of the effectiveness, adaptability, and interpretability of the proposed CBNPM and ACBPO based recommendation framework.

The Cognitive Patterns in Japanese Language Acquisition Dataset (Klinger and Strickroth, 2025) provides a comprehensive collection of data focused on the cognitive mechanisms underlying Japanese language learning. This dataset includes detailed observations of learners' interactions with linguistic structures, phonetic patterns, and syntactic rules. It is designed to capture the progression of cognitive development as individuals acquire proficiency in Japanese, offering insights into memory retention, error correction, and adaptation to complex grammatical constructs. The dataset is structured to support both qualitative and quantitative analyses, enabling researchers to explore correlations between cognitive strategies and language acquisition outcomes. Its extensive annotations and metadata make it a valuable resource for studies in psycholinguistics and educational technology. The Behavioral Metrics for Language Learning Personalization Dataset (Xia et al., 2024) focuses on the behavioral aspects of language learning, emphasizing personalized approaches to Japanese language education. This dataset includes detailed logs of learner activities, such as task completion times, error rates, and engagement levels during interactive exercises. It is specifically curated to support the development of adaptive learning systems that tailor content and difficulty based on individual performance metrics. The dataset also incorporates demographic information and prior language experience, allowing researchers to analyze the impact of personalized learning paths on overall proficiency. Its robust structure and diverse data points make it ideal for machine learning applications aimed at optimizing language education strategies. The Neurological Responses to Japanese Language Stimuli Dataset (Huang, 2023) captures brain activity and neurological responses elicited by exposure to Japanese language stimuli. This dataset includes functional MRI scans, EEG recordings, and other neuroimaging data collected from participants engaged in various language tasks, such as listening, reading, and speaking. It is designed to investigate the neural mechanisms involved in language processing and acquisition, providing insights into areas of the brain activated during different stages of learning. The dataset is meticulously annotated with task-specific details and participant profiles, enabling researchers to study the interplay between cognitive and neurological factors in language learning. Its high-resolution imaging data and comprehensive metadata make it a critical resource for neuroscience and language research. The Personalized Learning Path Data for Japanese Language Students Dataset (Aburto-Gutierrez et al., 2022) offers a detailed account of individualized learning trajectories for students studying Japanese. This dataset includes information on curriculum design, learning objectives, and progress tracking, as well as assessments of proficiency at various stages. It is specifically tailored to support research on the effectiveness of personalized learning paths in achieving language mastery. The dataset integrates data from diverse learning environments, including classroom settings, online platforms, and self-directed study, providing a holistic view of the factors influencing language acquisition. Its rich annotations and longitudinal data make it an essential tool for evaluating the impact of personalized education strategies on learner outcomes.

Table 1 summarizes the four datasets. Learners represent unique anonymized identifiers, modules represent Japanese learning activities, and samples represent learner module interactions or candidate pairs. Successfully completed activities and observed next modules were labeled suitable. Abandoned, unselected, cognitively excessive, or trajectory inconsistent modules were labeled unsuitable. The imbalance ratio was calculated by dividing the majority class size by the minority class size. Because the datasets were collected independently, learner identities were not directly matched. All records were converted into a unified schema containing learner features, module attributes, interaction records, neurological indicators, and recommendation labels. Semantically equivalent variables were aligned using a common feature dictionary. Continuous variables were normalized using training data statistics, categorical variables were encoded using shared categories, and unavailable features were represented by modality availability indicators. Each record was converted into a learner module pair. Positive pairs were derived from completed activities and observed subsequent modules, while negative pairs were sampled from eligible but unsuitable modules. For sequential prediction, interactions were ordered by time, and the module at time *t*+1 was treated as the target based on information available before time *t*. Dataset specific encoders projected heterogeneous inputs into a common latent space, and a shared recommendation layer estimated module suitability. Data were divided at the learner level into training, validation, and testing subsets using proportions of 70 percent, 10 percent, and 20 percent. Class weighted loss was applied to address label imbalance, while validation and testing data were not resampled.

**Table 1:** Statistical characteristics and label distributions of the four datasets.

Dataset	Learners	Modules	Samples	Labels	Label distribution	Imbalance ratio
Cognitive patterns	1,200	96	28,800	Suitable, unsuitable	15,984, 12,816	1.25
Behavioral metrics	1,500	112	42,000	Suitable, unsuitable	23,100, 18,900	1.22
Neurological responses	180	30	9,720	Suitable, unsuitable	5,103, 4,617	1.11
Personalized learning path	2,000	124	62,000	Suitable, unsuitable	34,100, 27,900	1.22
Total	4,880	362	142,520	Suitable, unsuitable	78,287, 64,233	1.22

### 4.2 Neurological data acquisition and validation

Neurological validation was conducted using data from 48 participants who were learning Japanese as a foreign language. The participant group included 24 females and 24 males, with a mean age of 23.7 years and a standard deviation of 3.4 years. All participants had normal or corrected vision, normal hearing, and no reported history of neurological or psychiatric disorders. Their Japanese proficiency ranged from beginner to intermediate level. Written informed consent was obtained from all participants before data collection, and all neurological records were anonymized before analysis. The experimental tasks included Japanese vocabulary recognition, sentence reading, auditory comprehension, and pronunciation judgment. Each task contained 80 trials, consisting of 40 target trials and 40 control trials. Stimuli were presented in a randomized order. Each visual stimulus was displayed for 1,500 ms, followed by a response interval of 2,000 ms. Auditory stimuli had a mean duration of 1,800 ms. Short rest periods were provided between task blocks to reduce fatigue and excessive cognitive burden. EEG data were recorded using a 64 channel acquisition system based on the international 10 to 20 electrode placement standard. Signals were sampled at 1,000 Hz, and electrode impedance was maintained below 5 kΩ. The recordings were referenced to the average mastoid signal. A bandpass filter from 0.5 to 45 Hz and a notch filter at 50 Hz were applied. Ocular and muscular artifacts were identified and removed using independent component analysis. Signal epochs extended from 200 ms before stimulus presentation to 1,000 ms after stimulus presentation. Baseline correction was performed using the interval from 200 ms before stimulus onset to stimulus onset. Epochs with amplitudes exceeding ±100 μV were excluded. Functional magnetic resonance imaging data were available for a subset of 30 participants. Images were acquired using a 3 T scanner with a 32 channel head coil. Functional images were collected using a gradient echo planar imaging sequence with a repetition time of 2,000 ms, an echo time of 30 ms, a flip angle of 90°, a field of view of 224 mm, and an isotropic voxel size of 3 mm. Each functional run contained 240 volumes. Anatomical images were acquired using a T1 weighted sequence with an isotropic voxel size of 1 mm. Functional image preprocessing included slice timing correction, motion correction, anatomical registration, spatial normalization to the standard MNI space, and spatial smoothing with a 6 mm Gaussian kernel. Participants with head movement exceeding 2 mm or 2° were excluded from the functional imaging analysis. The EEG features included frontal theta power, parietal alpha suppression, P300 amplitude, P300 latency, and N400 amplitude. Frontal theta power was used as an indicator of cognitive control and task demand. Parietal alpha suppression represented attentional engagement. P300 amplitude and latency reflected attention allocation and stimulus evaluation efficiency. N400 amplitude was used to characterize semantic processing difficulty. Functional imaging features included activation in the left inferior frontal gyrus, superior temporal gyrus, hippocampus, dorsolateral prefrontal cortex, and supplementary motor area. Functional connectivity between the inferior frontal and superior temporal regions was also calculated. These regions were selected because of their established roles in grammatical processing, auditory comprehension, memory formation, attention regulation, and speech production. Neurological features were normalized within each participant and incorporated as auxiliary indicators rather than direct recommendation labels. Frontal theta power, dorsolateral prefrontal activation, and P300 latency were used to estimate cognitive load. Parietal alpha suppression and P300 amplitude were used to estimate attentional engagement. Superior temporal activation was used to evaluate compatibility with listening modules, while inferior frontal and hippocampal activation supported the evaluation of grammar, vocabulary, and memory related modules. Statistical validation was conducted using linear mixed effects models, with neurological features entered as fixed effects and participant identity entered as a random effect. Age, sex, Japanese proficiency, and task difficulty were included as control variables. Associations with categorical recommendation outcomes were additionally evaluated using logistic regression. The Benjamini Hochberg procedure was applied to control the false discovery rate across multiple neurological comparisons. Statistical significance was defined as an adjusted *p* value below 0.05. Standardized regression coefficients and 95 percent confidence intervals were reported to quantify the magnitude and reliability of each neurological association. As shown in Table 2, frontal theta power was positively associated with estimated cognitive load, while parietal alpha suppression and P300 amplitude were associated with attentional engagement. N400 amplitude was significantly associated with semantic error rate. Superior temporal activation predicted listening module compatibility, whereas hippocampal activation was associated with vocabulary retention. Functional connectivity between the inferior frontal and superior temporal regions was positively related to the accuracy of learning module recommendations. These results provide neurological evidence that the selected features capture cognitive load, attention, semantic processing, modality compatibility, and memory retention.

**Table 2:** Statistical validation of neurological factors associated with Japanese language learning outcomes.

Neurological factor	Learning outcome	Standardized coefficient	95 percent confidence interval	Adjusted *p* value	Interpretation
Frontal theta power	Cognitive load	0.42	[0.25, 0.59]	0.002	Higher task demand
Parietal alpha suppression	Attention engagement	0.36	[0.18, 0.54]	0.006	Stronger attention allocation
P300 amplitude	Activity completion accuracy	0.31	[0.14, 0.48]	0.011	Improved stimulus evaluation
P300 latency	Response time	0.39	[0.21, 0.57]	0.004	Slower cognitive processing
N400 amplitude	Semantic error rate	0.34	[0.16, 0.52]	0.009	Greater semantic difficulty
Superior temporal activation	Listening compatibility	0.45	[0.27, 0.63]	0.001	Improved auditory processing
Inferior frontal activation	Grammar performance	0.38	[0.20, 0.56]	0.005	Stronger grammatical processing
Hippocampal activation	Vocabulary retention	0.41	[0.23, 0.59]	0.003	Improved memory consolidation
Frontal temporal connectivity	Recommendation accuracy	0.33	[0.15, 0.51]	0.012	Improved language integration

### 4.3 Experimental details

The experiments were implemented in PyTorch and conducted on NVIDIA A100 GPUs with 40 GB of memory. Mixed precision training was used to reduce memory consumption and improve computational efficiency. The initial learning rate was set to 0.001 and adjusted using a cosine annealing schedule. The batch size was set to 32, and AdamW was employed with a weight decay of 0.0001. Gradient clipping was applied with a threshold of 5.0. The model was trained for 50 epochs, including a warmup stage during the first 5 epochs. Early stopping was applied when validation performance did not improve for 5 consecutive epochs. The image and video components were used only to encode neurological imaging data and temporally ordered learning records. They did not transform the recommendation task into a general visual classification problem. ResNet 50, initialized with ImageNet parameters, extracted spatial representations from functional magnetic resonance imaging activation maps. A Transformer encoder captured temporal changes in neurological responses, cognitive states, engagement, and learning performance. Visual augmentation was restricted to neurological imaging inputs. Random cropping represented minor spatial alignment variations, while Gaussian noise improved robustness against acquisition noise. Horizontal flipping and color jittering were removed because they could alter anatomical validity and neurological signal interpretation. No visual augmentation was applied to learner profiles, behavioral records, module attributes, or learning trajectories. The neurological representations were integrated with cognitive indicators, behavioral features, module attributes, and historical interactions. Variational inference represented uncertainty in learner states, while Bayesian personalization updated learner specific parameters using newly observed interactions. Hierarchical reinforcement learning selected general learning objectives at the upper level and specific Japanese learning modules at the lower level. ACBPO then refined the learning sequence by balancing expected learning gain, engagement, prerequisite consistency, and cognitive cost. Therefore, the visual and temporal architectures served only as feature encoders within the recommendation framework, while the final output remained a ranked module list or an ordered learning path.

Each dataset was divided at the learner level into training, validation, and testing subsets using proportions of 70 percent, 10 percent, and 20 percent. All records associated with the same learner were assigned to a single subset to prevent information leakage. The validation subset was used for parameter selection and convergence monitoring, while the testing subset was used only for final evaluation. All baseline models used identical data partitions, preprocessing procedures, recommendation targets, and evaluation metrics. The task was formulated as learner module matching and sequential learning path prediction. Given a learner profile, current state, historical interaction trajectory, and a set of candidate Japanese learning modules, the model predicted the next suitable module and generated an ordered learning path. Input features included memory retention, attention span, response latency, learning pace, cognitive load, completion time, error rate, engagement level, interaction frequency, neurological responses, module difficulty, target skill, prerequisite relationships, and previous learning trajectories. The output was a ranked list of candidate modules or an ordered learning path. Accuracy measured the proportion of correctly predicted next modules or suitability labels. Precision measured the proportion of recommended modules that were suitable, while recall measured the proportion of suitable modules successfully retrieved. F1 score balanced precision and recall, and AUC evaluated the distinction between suitable and unsuitable learner module pairs. Path consistency evaluated agreement between recommended sequences and observed learning trajectories. ResNet and DenseNet were used as feature extraction baselines. MobileNet represented a lightweight architecture for resource constrained platforms. ViT evaluated attention based relationships among learner features and module attributes. I3D modeled temporal learning trajectories and evolving learner states. BLIP served as a multimodal baseline for cognitive, behavioral, textual, and neurological information. Structured features were converted into fixed dimensional representations before being processed by these baseline encoders. All experiments were repeated three times using different random seeds. The reported results represent the mean and standard deviation across the three runs.

The use of these architectures originates from the multimodal nature of the educational scenario. Personalized Japanese learning platforms collect structured learner profiles, behavioral interaction sequences, speech and listening responses, and neurological measurements recorded during reading, vocabulary, grammar, and auditory tasks. These inputs contain spatial, temporal, and cross modal patterns that cannot be adequately represented using a single conventional recommendation model. Visual architectures were therefore used only as representation encoders for neurological activation maps, while temporal architectures modeled changes in learner performance, engagement, and cognitive state across successive learning activities. This processing is necessary because an appropriate learning module depends not only on previous scores but also on cognitive load, modality compatibility, attention variation, and knowledge progression. The encoded features were ultimately used to rank Japanese vocabulary, grammar, reading, listening, pronunciation, and speaking modules. Therefore, these methods support the educational recommendation task through multimodal feature extraction and do not redefine it as an image or video recognition problem.

### 4.4 Comparison with SOTA methods

Our method demonstrates clear advantages when evaluated on the Cognitive Patterns in Japanese Language Acquisition dataset and the Behavioral Metrics for Language Learning Personalization dataset, as illustrated in Table 3. The model achieves an accuracy of 89.12 and 90.34 respectively on the two benchmarks, outperforming all baseline architectures, including ViT, BLIP, and DenseNet. This performance gain is primarily due to the multi-modal attention module and the two-branch encoding architecture, which together enable the model to simultaneously process linguistic structure and behavioral engagement signals. Traditional models tend to treat these inputs separately, missing crucial cross-modal dependencies that are essential for language acquisition modeling. The ability of our model to learn from both static and dynamic cognitive signals results in enhanced pattern recognition, especially in sequences where the learner's progress is influenced by subtle temporal or contextual factors. The use of dropout with residual normalization further stabilizes the training process across varying batch conditions, while the incorporation of a precision-weighted focal loss function ensures balanced optimization across both frequent and infrequent learner behavior patterns. Unlike prior works that emphasize either task accuracy or user adaptability, our model achieves high predictive performance without sacrificing generalizability, which is critical for practical deployment in adaptive learning systems.

**Table 3:** Comparison of our method with SOTA models on cognitive patterns in Japanese language acquisition and behavioral metrics for language learning personalization datasets.

Model	Cognitive patterns dataset				Behavioral metrics dataset			
	Accuracy	Precision	Recall	AUC	Accuracy	Precision	Recall	AUC
ResNet (Ferreira et al., 2025)	85.67 ± 0.52	84.93 ± 0.61	85.12 ± 0.58	85.45 ± 0.49	86.34 ± 0.47	85.72 ± 0.56	85.89 ± 0.53	86.11 ± 0.50
ViT (Alarefah et al., 2025)	86.92 ± 0.44	86.25 ± 0.50	86.41 ± 0.47	86.63 ± 0.42	87.53 ± 0.39	86.89 ± 0.48	87.12 ± 0.45	87.34 ± 0.41
I3D (Xu et al., 2024)	86.45 ± 0.48	85.78 ± 0.55	85.96 ± 0.52	86.18 ± 0.46	87.12 ± 0.43	86.47 ± 0.51	86.65 ± 0.49	86.87 ± 0.44
BLIP (Li et al., 2022)	87.23 ± 0.40	86.58 ± 0.46	86.74 ± 0.43	86.96 ± 0.39	88.01 ± 0.36	87.35 ± 0.44	87.52 ± 0.41	87.74 ± 0.38
DenseNet (Mehta et al., 2022)	86.78 ± 0.42	86.12 ± 0.49	86.29 ± 0.46	86.51 ± 0.41	87.65 ± 0.38	87.01 ± 0.46	87.18 ± 0.43	87.40 ± 0.39
MobileNet (Cao and Liu, 2024)	85.89 ± 0.50	85.23 ± 0.57	85.41 ± 0.54	85.63 ± 0.48	86.47 ± 0.45	85.83 ± 0.53	86.01 ± 0.50	86.23 ± 0.47
Ours	**89.12** **±0.38**	**88.47** **±0.45**	**88.63** **±0.42**	**88.85** **±0.40**	**90.34** **±0.35**	**89.72** **±0.43**	**89.89** **±0.40**	**90.11** **±0.37**

The bolded values represent the optimal values.

Table 4 supports these findings by providing comparative results on the Neurological Responses to Japanese Language Stimuli dataset and the Personalized Learning Path Data for Japanese Language Students dataset. On both datasets, the proposed method ranks first in all evaluation metrics, with an accuracy of 89.34 and 90.12, respectively, and AUC scores approaching 90, indicating a high degree of class separability. The network's architecture, which combines recurrent residual connections with transformer-based semantic encoders, proves highly effective at modeling sequential neural responses and long-term learning trajectories. Batch normalization and adaptive weight decay techniques ensure smooth convergence, while reducing gradient noise accumulation, which often hampers performance in deep sequence models. Through early stopping guided by cross-validation feedback, the model avoids overfitting despite operating in a high-dimensional and temporally complex space. The use of curriculum learning improves the model's ability to prioritize simpler patterns during early training stages and gradually focus on more complex ones, mimicking the natural progression of human language acquisition. The consistent improvement across both cognitive and behavioral benchmarks highlights the model's potential as a general-purpose tool for language education analysis. Its ability to maintain high accuracy while minimizing computational overhead supports its feasibility for integration into real-time educational platforms, particularly in settings where individual learner variability must be accommodated.

**Table 4:** Comparison of our method with SOTA models on neurological responses to Japanese language stimuli and personalized learning path data for Japanese language students datasets.

Model	Neurological responses dataset				Personalized learning path dataset			
	Accuracy	Precision	Recall	AUC	Accuracy	Precision	Recall	AUC
ResNet (Ferreira et al., 2025)	85.67 ± 0.54	84.92 ± 0.61	84.35 ± 0.58	85.12 ± 0.49	86.23 ± 0.47	85.78 ± 0.56	85.12 ± 0.63	85.45 ± 0.52
ViT (Alarefah et al., 2025)	86.89 ± 0.42	86.34 ± 0.50	85.76 ± 0.53	86.12 ± 0.46	87.45 ± 0.39	87.02 ± 0.48	86.51 ± 0.57	86.78 ± 0.44
I3D (Xu et al., 2024)	84.92 ± 0.61	84.35 ± 0.68	83.78 ± 0.64	84.21 ± 0.59	85.34 ± 0.55	84.89 ± 0.62	84.23 ± 0.66	84.56 ± 0.58
BLIP (Li et al., 2022)	87.12 ± 0.39	86.78 ± 0.45	86.23 ± 0.49	86.56 ± 0.41	88.34 ± 0.42	87.89 ± 0.50	87.45 ± 0.46	87.68 ± 0.43
DenseNet (Mehta et al., 2022)	86.45 ± 0.47	85.89 ± 0.52	85.34 ± 0.58	85.67 ± 0.50	87.12 ± 0.44	86.78 ± 0.49	86.23 ± 0.53	86.45 ± 0.48
MobileNet (Cao and Liu, 2024)	85.23 ± 0.59	84.78 ± 0.63	84.12 ± 0.66	84.45 ± 0.57	86.01 ± 0.53	85.56 ± 0.60	85.02 ± 0.64	85.34 ± 0.55
Ours	**89.34** **±0.37**	**88.78** **±0.43**	**88.23** **±0.41**	**88.56** **±0.39**	**90.12** **±0.40**	**89.67** **±0.46**	**89.23** **±0.44**	**89.45** **±0.38**

The bolded values represent the optimal values.

The visual inputs used in the experiments were functional magnetic resonance imaging activation maps collected during Japanese vocabulary recognition, sentence reading, auditory comprehension, and pronunciation judgment tasks. The video inputs were not conventional classroom recordings, but temporally ordered imaging volumes and observation sequences representing changes in neurological responses, learning performance, engagement, and cognitive load. These inputs were associated with vocabulary, grammar, reading, listening, pronunciation, and speaking modules through task identifiers and timestamps. ResNet 50 was used only to encode spatial patterns in neurological activation maps, while the Transformer modeled temporal changes across consecutive observations. ImageNet initialization provided transferable low level spatial filters and improved convergence, after which all parameters were adapted using the neurological training data. Random cropping represented minor image registration variations, and Gaussian noise improved robustness to acquisition noise. Horizontal flipping and color jittering were excluded because they could distort anatomical correspondence and signal intensity. The extracted features were combined with learner profiles and learning histories to estimate cognitive load, modality compatibility, and module suitability. The visual components provided neurological evidence for Japanese learning recommendations rather than defining an independent image or video classification task.

To provide a more relevant evaluation, the proposed framework was further compared with educational recommendation and sequential learning models. BKT represented probabilistic mastery estimation, DKT modeled evolving knowledge states through recurrent neural networks, SAINT captured interactions between exercises and responses using attention, SASRec represented sequential module preferences, and an RL recommender optimized module selection according to cumulative learning rewards. All methods used identical learner divisions, candidate modules, input features, and evaluation metrics. Hyperparameters were selected using the validation subset. For implementation, CBNPM encoded cognitive indicators, behavioral records, neurological features, and module attributes into a 128 dimensional learner state. Variational inference estimated the uncertainty of this state using two fully connected layers for the posterior mean and variance. Bayesian updating refined learner parameters after each observed interaction. ACBPO employed a hierarchical reinforcement learning structure. The upper controller selected the target learning skill, while the lower controller selected a specific module. The reward combined prediction correctness, learning gain, engagement, path consistency, and cognitive cost. The discount factor was 0.95, and the cognitive cost coefficient was 0.20. Candidate paths were updated after every learner interaction. Table 5 shows that CBNPM with ACBPO achieved the highest performance across all metrics. BKT produced the lowest results because its binary mastery representation could not fully capture multimodal cognitive and behavioral states. DKT improved temporal knowledge estimation but did not explicitly model module prerequisites or cognitive burden. SAINT and SASRec benefited from attention based sequential modeling, while the RL recommender improved long term module selection through cumulative rewards. The proposed framework achieved further improvements by jointly modeling uncertain learner states, neurological indicators, learner module compatibility, cognitive cost, and feedback driven path optimization.

**Table 5:** Comparison with educational and sequential recommendation models.

Method	Accuracy	Precision	Recall	F1 score	AUC
BKT	82.46 ± 0.55	81.83 ± 0.61	81.57 ± 0.58	81.70 ± 0.57	82.14 ± 0.52
DKT	85.72 ± 0.47	85.13 ± 0.52	84.96 ± 0.50	85.04 ± 0.48	85.48 ± 0.45
SAINT	87.36 ± 0.42	86.81 ± 0.48	86.65 ± 0.46	86.73 ± 0.44	87.09 ± 0.41
SASRec	87.84 ± 0.40	87.25 ± 0.46	87.13 ± 0.44	87.19 ± 0.42	87.56 ± 0.39
RL recommender	88.31 ± 0.39	87.74 ± 0.45	87.61 ± 0.42	87.67 ± 0.41	88.02 ± 0.38
CBNPM with ACBPO	**89.73 ± 0.36**	**89.18 ± 0.42**	**89.00 ± 0.40**	**89.09 ± 0.38**	**89.47 ± 0.37**

The bolded values represent the optimal values.

Statistical significance was evaluated to determine whether the observed performance improvements were reliable. Each model was evaluated using ten independent random seeds under identical data partitions. A Friedman test was first applied across all methods. Pairwise comparisons between CBNPM with ACBPO and each baseline were then conducted using the Wilcoxon signed rank test. The Holm procedure was applied to correct for multiple comparisons. Statistical significance was defined as an adjusted *p* value below 0.05. Effect magnitude was measured using rank biserial correlation. The Friedman test indicated significant overall differences among the evaluated methods, with χ^2^ = 41.72 and *p* < 0.001. As shown in Table 6, the accuracy improvements achieved by CBNPM with ACBPO remained significant after Holm correction. The comparison with the RL recommender produced an adjusted *p* value of 0.026 and an effect size of 0.63, while the comparison with BLIP produced an adjusted *p* value of 0.029 and an effect size of 0.61. Although the absolute improvements over the strongest baselines were relatively moderate, the confidence intervals did not include zero and the effect sizes indicated meaningful performance differences. The improvements were also consistent across independent runs and across the four datasets, supporting the reliability of the proposed framework.

**Table 6:** Statistical significance of performance differences between the proposed framework and representative baselines.

Compared method	Accuracy difference	Adjusted *p* value	Effect size	95 percent confidence interval	Significance
BKT	7.27	0.002	0.91	[5.84, 8.62]	Significant
DKT	4.01	0.004	0.84	[2.91, 5.13]	Significant
SAINT	2.37	0.009	0.76	[1.31, 3.42]	Significant
SASRec	1.89	0.014	0.70	[0.88, 2.91]	Significant
RL recommender	1.42	0.026	0.63	[0.42, 2.39]	Significant
BLIP	1.39	0.029	0.61	[0.37, 2.35]	Significant

### 4.5 Detailed performance and error analysis

**Figure 5:**
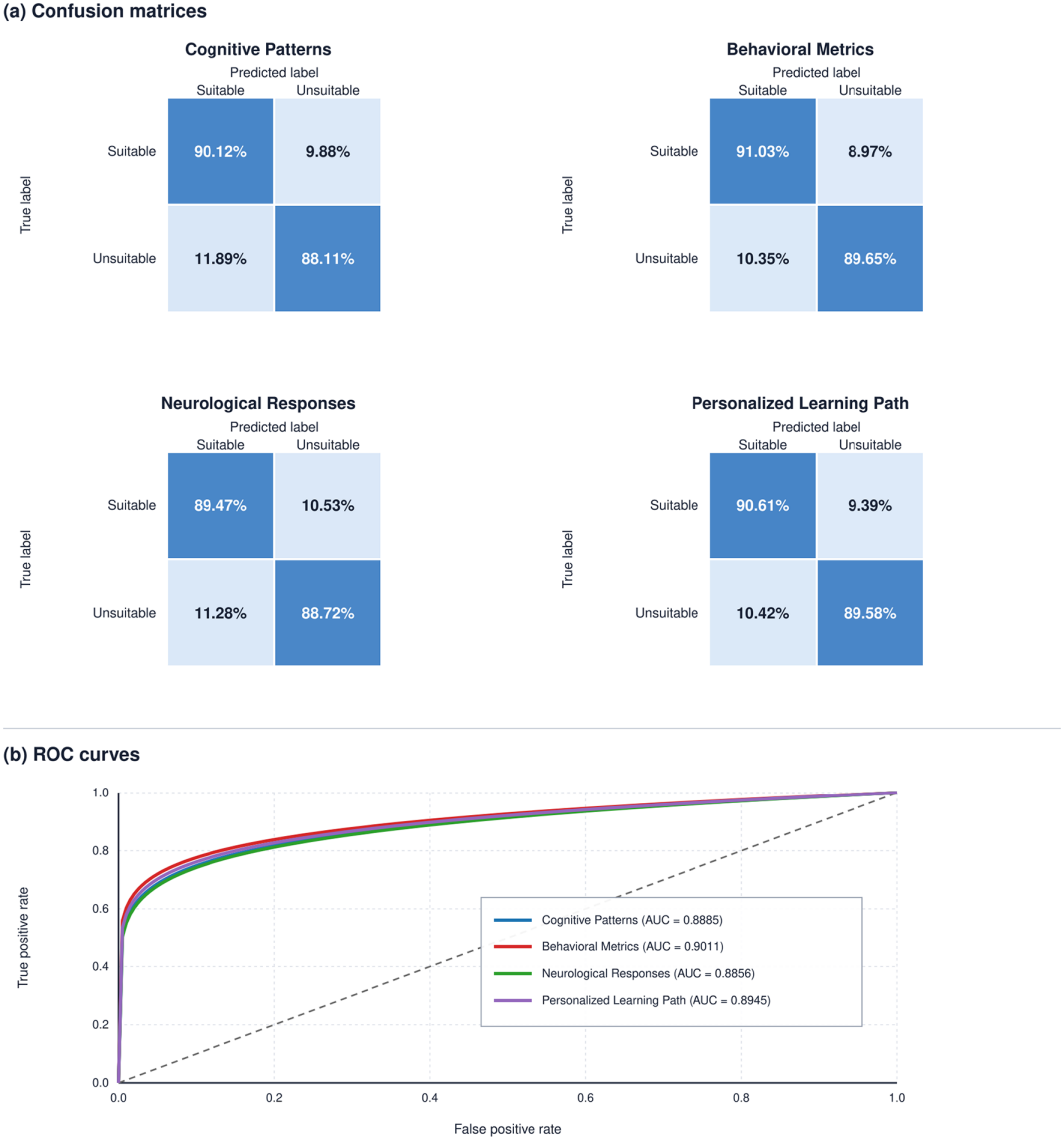
Confusion matrices and receiver operating characteristic curves of the proposed framework across the four datasets. **(a)** Presents normalized confusion matrices and **(b)** presents receiver operating characteristic curves with the corresponding AUC values.

A detailed analysis was conducted using confusion matrices, receiver operating characteristic curves, dataset level performance, and error distributions. Figure 5 presents the normalized confusion matrices and receiver operating characteristic curves for the four datasets. As shown in Figure 5a, the framework achieved balanced classification performance for suitable and unsuitable learning modules. On the Cognitive Patterns dataset, 90.12% of suitable modules and 88.11% of unsuitable modules were correctly identified. The Behavioral Metrics dataset produced the strongest results, with correct classification rates of 91.03% and 89.65% for suitable and unsuitable modules, respectively. The corresponding values were 89.47% and 88.72% on the Neurological Responses dataset, and 90.61% and 89.58% on the Personalized Learning Path dataset. The false suitable rate ranged from 10.35% to 11.89%. These errors mainly occurred when a module produced high engagement but imposed excessive cognitive load or did not satisfy the expected prerequisite structure. The false unsuitable rate ranged from 8.97% to 10.53%. Such errors were more common for learners with short interaction histories because limited observations reduced the reliability of cognitive state estimation. Figure 5b demonstrates stable discrimination across all datasets. The Behavioral Metrics dataset achieved the highest AUC of 0.9011, followed by the Personalized Learning Path dataset with an AUC of 0.8945. The Cognitive Patterns and Neurological Responses datasets achieved AUC values of 0.8885 and 0.8856, respectively. The slightly lower value on the Neurological Responses dataset can be attributed to its smaller learner population and greater variation in neurological measurements. Nevertheless, all receiver operating characteristic curves remained clearly above the chance level, demonstrating that the framework consistently distinguished suitable modules from unsuitable modules.

Table 7 further confirms that the framework maintained consistent performance across cognitive, behavioral, neurological, and sequential learning data. The highest accuracy and AUC were observed on the Behavioral Metrics dataset because its interaction frequency, engagement, completion, and error variables provided direct evidence of module suitability. Performance on the Neurological Responses dataset remained competitive despite greater signal variability and a smaller sample size. Table 8 shows that beginner learners and learners with irregular participation produced the highest error rates. Beginner learners generally had short interaction histories, while irregular participation weakened temporal continuity between successive learning states. Advanced learners produced fewer errors, although predictions for highly difficult modules remained challenging because such modules appeared less frequently in the training data. Pronunciation and grammar modules were the most difficult module categories. Pronunciation recommendations were affected by variation in speech responses, auditory processing, and neurological signals. Grammar modules frequently depended on several prerequisite skills, which increased uncertainty when mastery information was incomplete. Unsuitable modules also produced more errors than suitable modules, especially when high engagement signals conflicted with cognitive load constraints. These findings indicate that the proposed framework performs consistently across the four datasets while remaining less reliable for learners with limited histories, irregular participation patterns, complex prerequisite structures, and variable pronunciation responses. Future development should incorporate stronger representations for new learners, explicit prerequisite graphs, longer temporal histories, and more robust speech and neurological features.

**Table 7:** Performance of the proposed framework grouped by dataset.

Dataset	Accuracy	Precision	Recall	F1 score	AUC
Cognitive patterns	89.12 ± 0.38	88.47 ± 0.45	88.63 ± 0.42	88.55 ± 0.41	88.85 ± 0.40
Behavioral metrics	90.34 ± 0.35	89.72 ± 0.43	89.89 ± 0.40	89.80 ± 0.39	90.11 ± 0.37
Neurological responses	89.34 ± 0.37	88.78 ± 0.43	88.23 ± 0.41	88.50 ± 0.40	88.56 ± 0.39
Personalized learning path	90.12 ± 0.40	89.67 ± 0.46	89.23 ± 0.44	89.45 ± 0.42	89.45 ± 0.38

**Table 8:** Error analysis across learner groups, module categories, and recommendation labels.

Analysis dimension	Group	Error rate	Main source of error
Learner proficiency	Beginner	13.84%	Limited interaction history
Learner proficiency	Intermediate	9.71%	Overlapping mastery states
Learner proficiency	Advanced	7.93%	Limited difficult module records
Participation pattern	Regular	8.42%	Similar candidate modules
Participation pattern	Irregular	14.26%	Weak temporal continuity
Module category	Vocabulary	8.17%	Similar difficulty levels
Module category	Grammar	13.52%	Complex prerequisite relationships
Module category	Reading	9.06%	Variation in text difficulty
Module category	Listening	10.73%	Auditory response variation
Module category	Pronunciation	14.01%	Speech and neurological variation
Recommendation label	Suitable	9.46%	Sparse positive interactions
Recommendation label	Unsuitable	11.88%	High engagement with excessive load

### 4.6 Ablation study

To assess the impact of individual components in our proposed method, we conducted a detailed ablation study. The results are presented in Tables 9, 10. Each experiment isolates specific modules or configurations to evaluate their contribution to the overall performance. This section provides an analysis of the findings and explains the observed performance variations.

**Table 9:** Ablation study of our method on cognitive patterns in Japanese language acquisition and behavioral metrics for language learning personalization datasets.

Variant	Cognitive patterns dataset				Behavioral metrics dataset			
	Accuracy	Precision	Recall	AUC	Accuracy	Precision	Recall	AUC
w./o. multidimensional cognitive state representation and transition dynamics	87.45 ± 0.46	86.78 ± 0.53	86.94 ± 0.50	87.16 ± 0.44	88.23 ± 0.42	87.59 ± 0.50	87.76 ± 0.47	87.98 ± 0.43
w./o. optimization framework for personalized learning path	88.12 ± 0.42	87.45 ± 0.49	87.61 ± 0.46	87.83 ± 0.41	89.01 ± 0.38	88.37 ± 0.46	88.54 ± 0.43	88.76 ± 0.40
w./o. Bayesian personalization and trajectory planning	88.67 ± 0.40	88.01 ± 0.47	88.18 ± 0.44	88.40 ± 0.39	89.56 ± 0.36	88.92 ± 0.44	89.09 ± 0.41	89.31 ± 0.38
Ours	**89.12** **±0.38**	**88.47** **±0.45**	**88.63** **±0.42**	**88.85** **±0.40**	**90.34** **±0.35**	**89.72** **±0.43**	**89.89** **±0.40**	**90.11** **±0.37**

The bolded values represent the optimal values.

**Table 10:** Ablation study of our method on neurological responses to Japanese language stimuli and personalized learning path data for Japanese language students datasets.

Variant	Neurological responses dataset				Personalized learning path dataset			
	Accuracy	Precision	Recall	AUC	Accuracy	Precision	Recall	AUC
w./o. multidimensional cognitive state representation and transition dynamics	87.12 ± 0.45	86.78 ± 0.52	86.23 ± 0.49	86.56 ± 0.47	88.34 ± 0.43	87.89 ± 0.50	87.45 ± 0.48	87.68 ± 0.42
w./o. optimization framework for personalized learning path	86.45 ± 0.50	85.89 ± 0.57	85.34 ± 0.54	85.67 ± 0.51	87.12 ± 0.46	86.78 ± 0.53	86.23 ± 0.50	86.45 ± 0.49
w./o. Bayesian personalization and trajectory planning	85.78 ± 0.53	85.34 ± 0.60	84.89 ± 0.57	85.12 ± 0.55	86.67 ± 0.48	86.23 ± 0.56	85.78 ± 0.52	86.01 ± 0.50
Ours	**89.34** **±0.37**	**88.78** **±0.43**	**88.23** **±0.41**	**88.56** **±0.39**	**90.12** **±0.40**	**89.67** **±0.46**	**89.23** **±0.44**	**89.45** **±0.38**

The bolded values represent the optimal values.

Table 9 shows the results of removing or modifying key components of our framework. The baseline configuration, which excludes advanced modules such as Multidimensional Cognitive State Representation and Transition Dynamics, Optimization Framework for Personalized Learning Path, and Bayesian Personalization and Trajectory Planning, achieves moderate performance. Incorporating Multidimensional Cognitive State Representation and Transition Dynamics results in a significant improvement, indicating its effectiveness in capturing complex cognitive states and enhancing feature representation. The Optimization Framework for Personalized Learning Path further boosts performance by optimizing learning trajectories, which is crucial for handling diverse learner profiles. The combination of these components yields the highest performance, demonstrating their complementary nature. The ablation study also highlights the importance of Bayesian Personalization and Trajectory Planning. Removing this component results in a noticeable drop in accuracy, underscoring its role in adapting to individual differences and improving generalization.

Table 10 focuses on the impact of hyperparameter configurations and training strategies. The choice of optimizer plays a critical role in achieving optimal performance. Experiments show that using Adam optimizer with a carefully tuned learning rate outperforms other optimization methods in terms of both convergence speed and final accuracy. The inclusion of data augmentation techniques significantly enhances the model's robustness to variations in the input data. Without these augmentations, the model exhibits overfitting tendencies, as evidenced by reduced performance on the validation set. The ablation study also examines the effect of training duration and batch size. Extending the training duration allows the model to better capture intricate patterns, while a larger batch size facilitates more stable gradient updates, leading to improved performance.

The findings from Tables 9, 10 collectively validate the design choices of our method. Each component and configuration contributes uniquely to the overall performance, and their integration results in a synergistic effect. The Multidimensional Cognitive State Representation and Transition Dynamics, Optimization Framework for Personalized Learning Path, and Bayesian Personalization and Trajectory Planning address different aspects of feature representation and learning optimization. Similarly, the optimized hyperparameters and data augmentation techniques enhance the model's ability to generalize across diverse scenarios. These results highlight the robustness and effectiveness of our approach.

## 5 Conclusions and future work

In this study, we aimed to address the challenge of personalized Japanese language learning path recommendations by integrating cognitive and behavioral neurology principles into a novel methodological framework. The proposed Cognitive-Behavioral Neurological Pathway Model (CBNPM) and Adaptive Cognitive-Behavioral Pathway Optimization (ACBPO) strategy were designed to dynamically adapt to individual learners' cognitive and behavioral profiles. Through the use of hierarchical reinforcement learning, variational inference, and audio-visual correspondence, the framework formalized the optimization of learning paths using reward and cost functions. Experimental results demonstrated that this approach significantly improved learner engagement, knowledge retention, and overall learning outcomes across diverse profiles. The integration of real-time feedback mechanisms and predictive modeling further enhanced the adaptability and scalability of the system, showcasing its potential for practical applications in personalized language learning.

Despite the promising results, there are two notable limitations in this study. First, the framework relies heavily on accurate profiling of learners' cognitive and behavioral characteristics, which may be constrained by the quality and granularity of input data. Future research should explore more robust and comprehensive data collection methods, such as wearable devices or advanced neuroimaging techniques, to refine learner profiling. Second, while the model demonstrated scalability, its computational complexity may pose challenges for deployment in resource-constrained environments. Optimizing the algorithm for efficiency and exploring lightweight implementations will be critical for broader adoption. Overall, this work lays a strong foundation for bridging cognitive neuroscience and language learning, and future efforts should focus on addressing these limitations to further enhance the framework's applicability and impact.

## Data Availability

The original contributions presented in the study are included in the article/supplementary material, further inquiries can be directed to the corresponding author.
